# Genome-Wide Screening Reveals the Oncolytic Mechanism of Newcastle Disease Virus in a Human Colonic Carcinoma Cell Line

**DOI:** 10.3390/v17081043

**Published:** 2025-07-25

**Authors:** Yu Zhang, Shufeng Feng, Gaohang Yi, Shujun Jin, Yongxin Zhu, Xiaoxiao Liu, Jinsong Zhou, Hai Li

**Affiliations:** 1 Department of Histology and Embryology, School of Basic Medical Sciences, Xi’an Jiaotong University, Xi’an 710061, China; yuzhang@cau.edu.cn (Y.Z.); jinshujun0313@stu.xjtu.edu.cn (S.J.); 2 Department of Pathogenic Biology and Immunology, School of Basic Medical Sciences, Xi’an Jiaotong University, Xi’an 710061, China; feng_xjtu0204@163.com (S.F.); 1605094195@stu.xjtu.edu.cn (G.Y.); zyx2023@stu.xjtu.edu.cn (Y.Z.); liuxiaoxiao12@stu.xjtu.edu.cn (X.L.); 3 College of Animal Science and Technology, Guangxi University, Nanning 530004, China; 4 Department of Medical Engineering, School of Future Technology, Xi’an Jiaotong University, Xi’an 710049, China

**Keywords:** oncolytic virus, colonic carcinoma, Newcastle disease virus, CRISPR screening, RNA sequencing

## Abstract

Viral oncolysis is considered a promising cancer treatment method because of its good tolerability and durable anti-tumor effects. Compared with other oncolytic viruses, Newcastle disease virus (NDV) has some distinct advantages. As an RNA virus, NDV does not recombine with the host genome, making it safer compared with DNA viruses and retroviruses; NDV can induce syncytium formation, allowing the virus to spread among cells without exposure to host neutralizing antibodies; and its genome adheres to the hexamer genetic code rule (genome length as a multiple of six nucleotides), ensuring accurate replication, low recombination rates, and high genetic stability. Although wild-type NDV has a killing effect on various tumor cells, its oncolytic effect and working mechanism are diverse, increasing the complexity of generating engineered oncolytic viruses with NDV. This study aims to employ whole-genome CRISPR-Cas9 knockout screening and RNA sequencing to identify putative key regulatory factors involved in the interaction between NDV and human colon cancer HCT116 cells and map their global interaction networks. The results suggests that NDV infection disrupts cellular homeostasis, thereby exerting oncolytic effects by inhibiting cell metabolism and proliferation. Meanwhile, the antiviral immune response triggered by NDV infection, along with the activation of anti-apoptotic signaling pathways, may be responsible for the limited oncolytic efficacy of NDV against HCT116 cells. These findings not only enhance our understanding of the oncolytic mechanism of NDV against colonic carcinoma but also provide potential strategies and targets for the development of NDV-based engineered oncolytic viruses.

## 1. Introduction

Oncolytic viruses (OVs) are capable of infecting and killing human tumor cells, yet they have no cytotoxic effect on normal cells. Due to their good tolerability and durable anti-tumor effects, viral oncolysis is supposed to be a promising cancer treatment method. Since the 1990s, genetic engineering techniques have been applied to modify viruses for enhanced oncolytic efficacy. Notable examples include ONYX-015 (dl1520), a modified adenovirus type 5 with deletions in the E1B-55k gene that restrict its replication to p53-deficient cancer cells [1], and talimogene laherparepvec (T-VEC), a genetically modified herpes simplex virus 1 (HSV-1) engineered to delete the *ICP34.5* gene (reducing neurovirulence) and express GM-CSF (enhancing systemic antitumor immune responses) [2]. These advances have substantially accelerated research and development efforts for such viruses. To date, only four oncolytic virus drugs have been approved for clinical use: RIGVIR (an ECHO-7 virus) for melanoma, Oncorine (H101, an adenovirus) for nasopharyngeal carcinoma, T-VEC (HSV) for metastatic melanoma, and Teserpaturev (G47Δ HSV) for malignant glioblastoma. Beyond these, numerous OVs—encompassing both DNA and RNA viruses—are in clinical trials. These include wild-type viruses with natural oncolytic properties as well as genetically engineered oncolytic viruses [3,4]

Historically, researchers regarded direct tumor cell killing as the sole anti-tumor mechanism of OVs, with the host immune system considered a major barrier to OV therapy. However, recent preclinical and clinical studies have revealed that the immune response actually enhances OV therapeutic efficacy, as these viruses induce and potentiate the body’s anti-tumor immunity [5,6]. Consequently, the primary emphasis of oncolytic virus research has shifted from the direct cytotoxic impact of viruses on tumors to the induction of immune responses within the body to eliminate tumor cells. This shift has driven efforts to combine OVs with immunotherapy to achieve synergistic effect. A prime example is T-VEC, the first oncolytic agent approved by the FDA and European regulatory authorities in 2015. Beyond its direct oncolytic activity, T-VEC encodes the *GM-CSF* gene, which recruits and activates host immune cells [7]. In the Phase III OPTIM trial, T-VEC achieved a 16.3% durable response rate, compared to 2.1% with GM-CSF alone, underscoring the synergistic interplay of viral lysis and immune stimulation [2]. Nonetheless, optimizing direct oncolysis remains important, as it reduces reliance on immune activation, making therapy feasible even in immunologically “cold” tumors and in patients with cancer cachexia. Despite these advances, key challenges persist, including limited therapeutic efficacy, immune evasion, tumor heterogeneity, and hurdles in viral delivery. These obstacles impede the clinical scalability of oncolytic virus therapy (OVT) and the translational success of emerging candidates. Addressing these challenges during drug design and preclinical validation is critical to minimizing attrition in cancer drug development and advancing OVT toward broader clinical application.

As early as 1965, Cassel and Garret from Atlanta, USA, discovered the tumor-killing effect of Newcastle Disease Virus (NDV) [8]. Compared with other viruses, NDV has the following distinct advantages as an oncolytic agent: although NDV is infectious and pathogenic in poultry, it exhibits minimal pathogenicity in humans, causing only mild symptoms such as conjunctivitis and laryngitis upon infection [9]; NDV is an RNA virus that does not undergo recombination with the host genome during replication within host cells, making it safer compared with DNA viruses and retroviruses [10]; NDV can induce syncytium formation, causing infected cells to fuse with neighboring cells to form large multinucleated cells, allowing the virus to spread among cells without exposure to host neutralizing antibodies [11]; the NDV genome adheres to hexamer genetic code rule, which dictates that the total number of nucleotides (nts) in the viral genome must be an exact multiple of six nucleotides. Deviations from the six nucleotides rule disrupt the precise alignment of the viral RNA-dependent RNA polymerase (L protein) with the nucleocapsid template, severely impairing RNA synthesis and viral propagation. The rule imposes strong selective pressure on viral genomes, limiting insertions/deletions to multiples of six nucleotides to maintain evolutionary constraint, resulting in an extremely low rate of gene recombination and high genetic stability [12,13].

When NDV infects normal cells, it is recognized by intracellular RIG-I-like receptors, triggering a strong interferon response that inhibits viral replication. However, in tumor cells, due to immune dysregulation, NDV replication is no longer restricted, enabling it to deplete and kill tumor cells [14]. Studies have shown that overexpression of RIG-I in tumor cells weakens NDV’s killing effect on tumor cells [15]. Additionally, Caspase-dependent cell death pathway, p38-MAPK signaling pathway, and endoplasmic reticulum stress pathway also play important roles in the oncolytic process of NDV [16,17]. Therefore, the possible mechanisms of NDV’s specific killing of tumor cells include the following: defects in the antiviral signaling pathway of tumor cells; defects in the type I interferon signaling pathway of tumor cells; defects in the apoptotic pathway of tumor cells [18]. In addition, NDV infection will elicit the release of certain cytokines and chemokines from tumor cells, thereby activating the body’s anti-tumor immune response and indirectly killing the tumor cells [19].

Although studies have shown that NDV has a killing effect on various types of tumor cells, its oncolytic effect and working mechanism are diverse [20]. Optimization of NDV’s oncolytic efficiency of is urgently needed but constrained by incomplete mechanistic understanding of host–virus interactions. This study aims to employ whole-genome CRISPR-Cas9 knockout screening and RNA sequencing to identify key regulatory factors involved in the interaction between NDV and human colon cancer cells and further investigate their interaction mechanism. The results will provide theoretical foundations for systematically interpreting the oncolytic mechanism of NDV against colonic carcinoma and for developing NDV-based engineered oncolytic viruses targeting colon cancer.

## 2. Materials and Methods

### 2.1. Cells, Plasmids, and Virus Strain

NDV LaSota, a low-pathogenicity vaccine strain, was generated using a standard reverse genetics system comprising a full-length genomic backbone plasmid (incorporating EGFP) and three helper plasmids (pVAX1-NP, pVAX1-P, and pcDNA-L) constructed based on the complete NDV LaSota genome sequence (GenBank: AY845400.2) and preserved in our laboratory. All viral operations were performed in a Biosafety Level 2 (BSL-2) facility in accordance with institutional biosafety guidelines. Viruses were propagated in BHK-21 cells and purified with HiScreen Capto Core 700 Column (MilliporeSigma, Burlington, MA, USA). Virus titers were determined in HCT116 cells via flow cytometric analysis of EGFP-positive cells. Briefly, cells were infected with 10-fold serial dilutions of viral supernatants (with three replicates per dilution) and analyzed at 24 h post-infection (hpi). Phosphate-buffered saline (PBS) was used as Mock. Infectious titers, expressed as transducing units per milliliter (TU/mL), were calculated using the formula: Titer (TU/mL) = [Percentage of EGFP-positive cells × number of cells per well]/[Volume of viral inoculum (mL) × dilution factor].

Human CRISPR Knockout Pooled Library (GeCKO v2) (Pooled Libraries #1000000048) and plasmids lentiCRISPR v2 (Plasmid #52961), pCMV-VSV-G (Plasmid #8454), and psPAX2 (Plasmid #12260) were purchased from Addgene (Watertown, MA, USA).

Human colon cancer cell line HCT116 (CCL-247) was purchased from ATCC, USA. Human colon epithelial cell line NCM460 (Delf-14048), validated by lack of oncogenic mutations and normal karyotype, was purchased from Wanwu Biotechnology, China. The cells were cultured in Dulbecco’s modified eagle medium (DMEM) supplemented with 10% fatal bovine serum (FBS) (C0235, Gibco, Carlsbad, CA, USA) and 1% penicillin–streptomycin (C0222, Beyotime, Shanghai, China). The cells were maintained at 37 °C, 5% CO_2_ in a humidified atmosphere. All cells used in the present study were within passage 20.

### 2.2. Virus Infection

When the cells reached 80% confluency, the culture medium was aspirated, and the cells were washed with PBS. Then the cells were infected with NDV-LaSota at a multiplicity of infection (MOI) of 5 and further cultured in DMEM containing 5% FBS for 24 or 48 h. Samples were visualized using Nikon 80i fluorescence microscope (Nikon, Tokyo, Japan).

### 2.3. Absolute Quantitative PCR (qPCR)

To detect viral genome replication, viral RNA was extracted using Mini BEST Viral RNA/DNA Extraction Kit Ver. 5.0 (Takara Bio Inc., Beijing, China) according to the manufacturer’s protocol. Subsequent absolute quantitative PCR (qPCR) detection was performed using ABclonal 2X Universal SYBR Green Fast qPCR Mix (ABclonal, Wuhan, China) with an applied biosystem (Bioer Technology, Hangzhou, China) according to the manufacturer’s protocol. The following absolute qPCR primers targeting the fragment located between *M* and *F* genes of NDV were designed according to the complete genome of NDV LaSota strain (GenBank: AY845400.2): F:5′-ctgcgtctctgagattgcgc-3′, R: 5′-cttgcacctggagggcg-3′. The standers of absolute qPCR were prepared by cloning the PCR products into the pMD™18-T Vector (Takara Bio Inc., Beijing, China) according to the manufacturer’s instruction.

### 2.4. Flow Cytometric Analysis

Cells at 80% confluency were infected with NDV at a MOI of 5 and cultured in DMEM with 5% FBS for 24 or 48 h. Supernatants and trypsinized cells were collected, centrifuged (500× *g*, 5 min), and washed with PBS. For apoptosis analysis, cells were resuspended in Annexin V Binding Buffer and stained with PE Annexin V and 7-AAD at room temperature in the dark for 15 min follow manufacturers’ instructions (Solarbio, Beijing, China). For cell cycle detection, cells were collected at 24 hpi and fixed with 70% ethanol at −20 °C for 12 h. After washing with cold PBS, cells were stained with PI solution at room temperature in the dark for 20 min. Samples were detected on a NovoCyte Advanteon (Agilent, Hangzhou, China) and analyzed with FlowJo software, v. 10.8.1 (BD Biosciences, Ashland, OR, USA).

### 2.5. RNA Sequencing

HCT116 cells were infected with NDV at a MOI of 5, and a control group without NDV infection was established. Then, 24 h after viral infection, genome-wide gene expression profiling of the cells was employed via RNA deep sequencing. RNA was isolated from cells using the RNeasy Plus mini kit (74314, QIAGEN, Hilden, Germany), with RNA integrity numbers (RIN) ranging from 8.4 to 9.8. Library construction was performed using the Illumina platform (Illumina, Inc., San Diego, CA, USA) according to the manufacturer’s instruction. The samples were sequenced on an Illumina HiSeq 2500 instrument, with average raw reads number of 56,483,404.5, average clean reads rate at 96.4%, average raw Q30 bases rate at 92.7%, and average clean Q30 bases rate at 93.04%. As for each group, four biological repeats were performed.

### 2.6. CRISPR-Cas9 Knockout Screening

Genome-wide CRISPR-Cas9 knockout (KO) screening was performed using a lentiviral library comprising 123,411 single-guide RNAs (sgRNAs) targeting 19,050 protein-coding human genes [21]. The lentiviral library infected HCT116 cells at a MOI of 0.4. DMEM containing the lentiviral library, and 5% FBS was added to the cell culture flasks along with 100 μL polybrene. After 12 h, the culture medium was discarded, and DMEM containing 5% FBS was added for continued culture. Then, 12 h later, puromycin at a final concentration of 1.5 μg/mL was added to the medium to screen for cells expressing both sgRNA and Cas9, and the control group was also treated with the same concentration of puromycin. Once all cells in the control group died, the surviving cells from the infected group were collected and passaged for further culture.

The screened cells were infected with NDV at a MOI of 5, and a control group without NDV infection was established. Subsequently, 48 h later, the surviving cells were collected and centrifuged at 2000× *g* for 20 min. The supernatant was discarded, and the cell genome was extracted according to the manufacturer’s instruction. The libraries were PCR-amplified for 16 cycles using Phusion Flash High-Fidelity (Thermo Fisher, Waltham, MA, USA) with the following primers (annealing temperature of 55 °C, an extension time of 30 s), to add adaptors for Illumina sequencing: Adaptor_F: AATGGACTATCATATGCTTACCGTAACTTGAAAGTATTTCG; Adaptor_R: TCTACTATTCTTTCCCCTGCACTGTTGTGGGCGATGTGCGCTCTG. Through PCR amplification, the sgRNA in the genome is amplified, and the PCR products were extracted with a Gel-extraction kit (Omega Bio-Tek Inc., Norcross, GA, USA) following electrophoresis. Then the sequencing library is subjected to next-generation high-throughput sequencing using the Illumina HiSeq 2500 platform.

### 2.7. High-Throughput Data Analysis

As for CRISPR-Cas9 knockout screening, the sequencing data were analyzed using R (v4.2.0) with package edgeR (v3.40.2). The matrix of counts returned by the processAmplicons function was subjected to data quality assessment and normalization using the calcNormFactors function. Statistical testing for changes in sgRNA abundance between NDV-infected and control groups were carried out using the exactTest function, which allows for results to be ranked by significance using the topTags function. A gene-by-gene ranking was obtained by summarizing data across multiple sgRNAs targeting the same gene using the camera gene-set test [22].

RNA sequencing data was analyzed with the Galaxy web-based tool [23] and R (v. 4.2.0). DNA reads were aligned to the GRCh38 human reference genome (Homo_sapiens. GRCh38.p8) and annotated with a Homo_sapiens. GRCh38.91.chr.gtf file. Differentially expressed genes (DEG) were identified using edgeR, and the thresholds were |log2-fold change (FC)| > 1.0 and *p* < 0.05 [24]. Gene ontology and pathway analysis was performed with R packages clusterProfiler (v. 4.6.2) and pathview (v. 1.38.0). Protein interaction information was acquired using the STRING database (https://cn.string-db.org/, accessed on 30 April 2025) [25]. RNA-seq raw data were uploaded to the National Center for Biotechnology Information database under the accession number GSE297244.

### 2.8. Statistical Analysis

The statistical significance of data between different groups was determined with GraphPad prism software (Version 5.01, GraphPad Software Inc., La Jolla, CA, USA). All data were expressed as mean ± SD; significance was determined by performing one- or two-sided Student’s *t*-tests and defined as *p* value < 0.05.

## 3. Results

### 3.1. Oncolytic Effects of NDV on Different Cells

**Figure 1:**
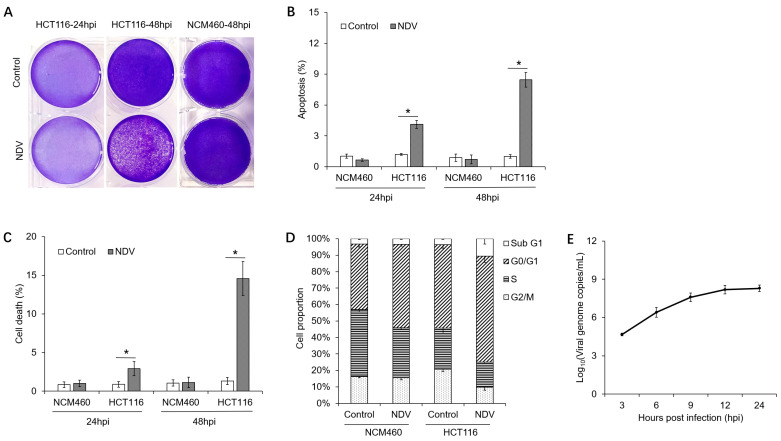
Characterization of the killing effects of NDV on different cells. (**A**) Cellular morphologies were observed after 24 or 48 h of NDV infection at a MOI of 5. Cells were stained with crystal violet. (**B**,**C**) The ratios of apoptotic and dead cells were evaluated via flow cytometry after 24 or 48 h of NDV infection. mean ± SD. n = 3. The bars labeled with asterisk are significantly different (*p* < 0.05). (**D**) The proportions of cells within different cell cycle phases were compared at 24 h post NDV infection. (**E**) Viral genome replication of NDV in HCT116 cells was determined by absolute quantitative PCR. mean ± SD. n = 3. *, *p* < 0.05.

Human colon epithelial cells (NCM460) and colon cancer cells (HCT116) were infected with NDV-LaSota at a MOI of 5, and the cellular morphology was observed after 24 and 48 h. As shown in Figure 1A, after 24 h of viral infection, dead cells were observed in HCT116 cultures, and by 48 h, a large portion of HCT116 cells had detached and died. In contrast, NCM460 cells did not exhibit significant morphological changes within 48 h of NDV infection.

Flow cytometry was employed to evaluate the alterations in the ratios of dead and apoptotic cells following NDV infection. As shown in Figure 1B,C, 24 h after viral infection, the rate of apoptosis in HCT116 cells was 4.12 ± 0.38%, and the rate of cell death was 2.92 ± 0.93%, both significantly higher than the control group’s 1.29 ± 0.09% and 0.87 ± 0.38%. After 48 h of infection, the apoptosis rate in HCT116 cells increased to 8.46 ± 0.71%, and the cell death rate rose to 14.59 ± 2.19%, significantly surpassing the control group’s 1.00 ± 0.18% and 1.31 ± 0.45%. During this period, no significant differences were observed in the rates of apoptosis and cell death between the NDV-treated NCM460 cells and the control cells.

Then, we compared the cell cycle profiles of the groups at 24 h post NDV infection. The results are depicted in Figure 1D and Figure S1. In the NDV-treated NCM460 cells, the proportion of cells in G0/G1 phase was 50.79 ± 1.02%, significantly higher than 39.79 ± 1.87% observed in the control group. Conversely, the proportion of cells in the S phase was 30.28 ± 1.21%, significantly lower than 40.70 ± 1.66% in the control group. No significant changes were observed in the proportions of cells in G2/M or subG1 phases. In the NDV-infected HCT116 cells, the proportion of cells in G0/G1 phase was 64.44 ± 3.78%, significantly higher than 50.73 ± 2.11% in the control group. The proportion of cells in the S phase was 15.12 ± 0.67%, significantly lower than 24.81 ± 2.90% in the control group. The proportion of cells in the G2/M phase was 9.88 ± 1.85%, significantly lower than 20.77 ± 1.47% in the control group, while the proportion of cells in the subG1 phase was 10.52 ± 3.19%, significantly higher than 3.62 ± 0.36% in the control group. These findings indicated that while NDV stimulation induced G1 phase arrest in NCM460 cells, it did not lead to apoptosis or cell death. In contrast, NDV infection not only caused G1 phase arrest in HCT116 cells but also triggered substantial apoptosis and cell death. Figure 1E demonstrates the increase in viral genome replication levels in HCT116 cells, as measured using absolute quantitative PCR.

### 3.2. Identification of the Putative Regulatory Factors Involved in NDV Oncolysis

CRISPR-Cas9 gene knockout library can be used for either positive selection or negative selection. In our study, positive selection identifies genes whose knockout enhances the cell’s ability to resist NDV infection, whereas negative selection identifies genes whose knockout promotes cell death following NDV infection. Given the oncolytic effects of NDV on HCT116 cells (Figure 1 and Figure S1), we analyzed both sets of genes. Using R (v4.2.0) with the edgeR package (v. 3.40.2) and a significance threshold of *p* < 0.05, we identified 906 differentially expressed genes in the NDV-treated HCT116 cells (Table S1). Among these, 425 genes were positive selection genes, while 481 genes were negative selection genes. These results suggest that the 425 positive selection genes may be putative regulators of host cells to respond to NDV oncolysis, whereas the 481 negative selection genes may play essential roles in defending NDV infection.

**Figure 2:**
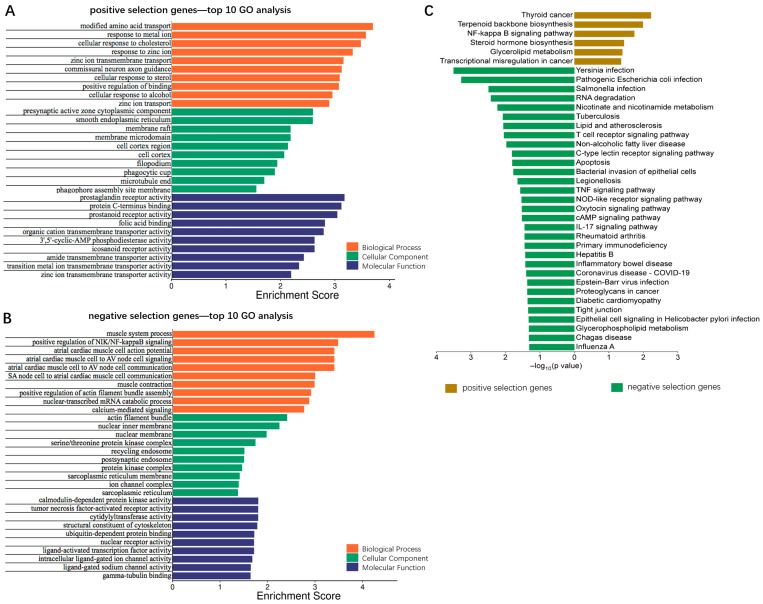
GO and KEGG pathway analysis of the putative regulatory factors involved in NDV oncolysis. (**A**,**B**) GO analysis was applied to (**A**) 425 positive and (**B**) 481 negative selection genes, and functional annotation was performed from the biological process, cellular component, and molecular function. The items were ranked in ascending order of *p*-value, and the top ten from each group were presented. (**C**) KEGG pathway analysis was applied to 425 positive and 481 negative selection genes. The items were ranked in ascending order of *p*-value.

Subsequently, we conducted gene ontology (GO) analysis on the two sets of genes. Functional annotation was performed from three perspectives, biological process, cellular component, and molecular function, and the results are illustrated in Figure 2A,B and Table S2. The positive selection genes were primarily involved in the amino acid and metal ion transport processes as well as the cellular responses to sterol, while the negative selection genes were mainly implicated in the regulation of NIK/NF-κB signaling and the compositions of actin filament and nuclear membrane. Then, we performed Kyoto Encyclopedia of Genes and Genomes (KEGG) pathway analysis on the positive and negative selection genes. As shown in Figure 2C and Table S3, the positive selection genes were predominantly enriched in the pathways related to cancer, NF-κB signaling and biosynthesis of terpenoid and steroid, among others, while the negative selection genes were mainly enriched in the pathways associated with infection, RNA degradation, and metabolism of nicotinate and lipids, among others.

**Figure 3:**
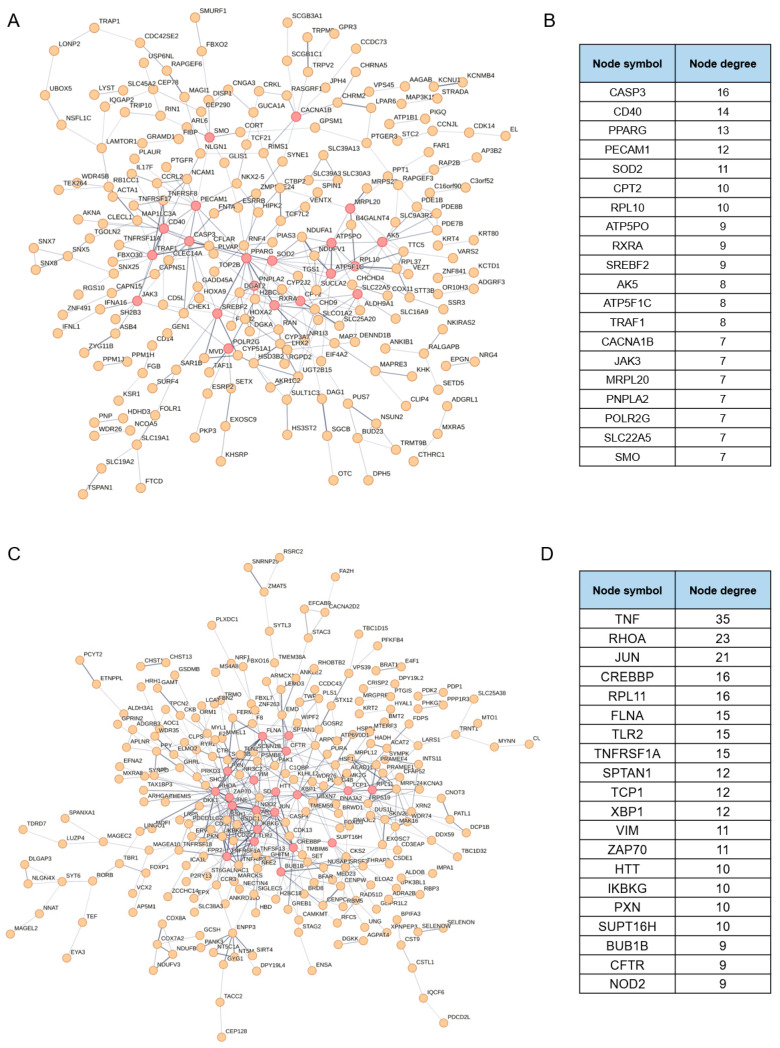
PPI analysis of the putative regulatory factors involved in NDV oncolysis. PPI networks of (**A**,**B**) 228 positive selection genes and (**C**,**D**) 258 negative selection genes with the top 20 ranked genes highlighted in red and listed separately. Proteins are represented as nodes with lines indicating a functional relationship.

Finally, we conducted protein–protein interaction (PPI) network analysis on the positive and negative selection genes to elucidate their interactions. As illustrated in Figure 3A,B, 228 out of 425 positive selection genes are associated with each other. Among the top 20 ranked genes, *CASP3*, *CD40*, and *TRAF1* are involved in regulating cellular survival or apoptosis signaling pathways; *CD40*, *JAK3*, and *TRAF1* collectively modulate immune and inflammatory reactions; *PPARG*, *RXRA*, *POLR2G*, and *RPL10* regulate gene transcription and protein synthesis; *PPARG*, *SREBF2*, *CPT2*, and *PNPLA2* play roles in regulating lipid metabolism; *SOD2* scavenges oxygen free radicals to sustain intracellular environmental stability; and *ATP5PO*, *ATP5F1C*, and *MRPL20* are implicated in mitochondrial ATP production and protein synthesis. Thus, these proteins act synergistically in cellular survival, energy metabolism, immune response, and stress protection, contributing to the maintenance of homeostasis. Among the 481 genes identified in negative selection, 258 were functionally associated with one another. Notably, among the top 20 ranked genes, *TNF*, *TLR2*, *TNFRSF1A*, *NOD2*, *ZAP70*, and *IKBKG* play pivotal roles in pathogen recognition, immune activation, and inflammatory regulation. The transcription factors *JUN*, *CREBBP*, *XBP1*, and the chromatin transcription regulator *SUPT16H* govern gene transcription. *FLNA*, *SPTAN1*, *VIM*, and *PXN* collectively participate in cytoskeletal organization, cell adhesion and signal transduction, forming a structural-functional network essential for cellular integrity. *RPL11* collaborates with *TCP1* to orchestrate protein synthesis and folding, thereby maintaining proteostasis. Additionally, *BUB1B* safeguards cell cycle progression through checkpoint surveillance; *XBP1* mediates the unfolded protein response under ER stress; *CFTR* regulates ion homeostasis across epithelial barriers; and *HTT* coordinates intracellular trafficking. Collectively, these proteins respond to cellular stressors by modulating immune defense, gene expression, protein metabolism, cellular architecture, and cell cycle regulation, and their dysregulation may result in immunological disorders or metabolic dysfunction.

### 3.3. Identification of the Putative Effectors Involved in NDV Oncolysis

**Figure 4:**
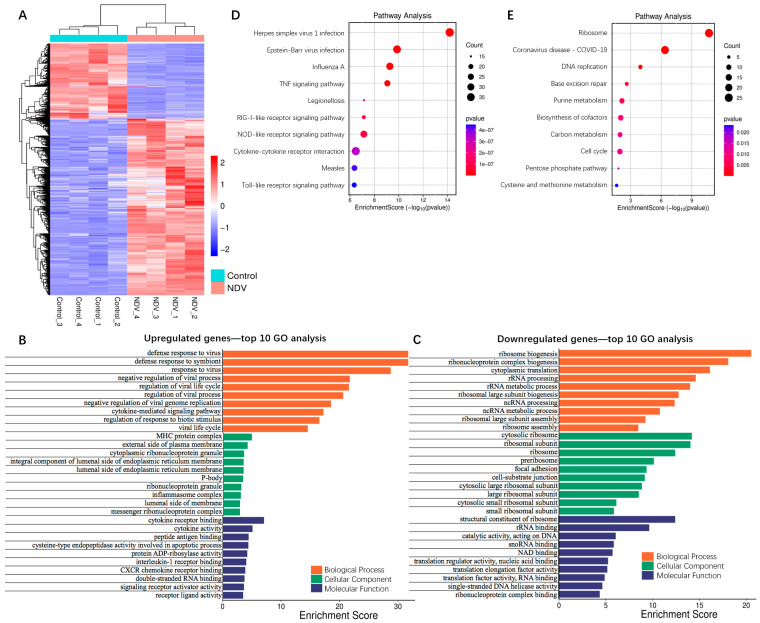
GO and KEGG pathway analysis of the putative effectors involved in NDV oncolysis. (**A**) Hierarchical clustering analysis of the genes differentially expressed between NDV-treated cells and control cells at *p* < 0.05, |log_2_ fold change| > 1.0. Columns indicate samples and rows indicate genes. Blue indicates repression and red indicates promotion. (**B**,**C**) GO analysis was applied to (**B**) 1402 upregulated genes and (**C**) 593 downregulated genes, and functional annotation was performed from the biological process, cellular component, and molecular function. The items were ranked in ascending order of *p*-value, and the top ten from each group were presented. (**D**,**E**) KEGG pathway analysis was applied to (**D**) 1402 upregulated genes and (**E**) 593 downregulated genes. The items were ranked in ascending order of *p*-value, and the top ten from each group were presented.

Genome-wide gene expression profile of HCT116 cells was conducted 24 h post NDV infection using RNA sequencing. As illustrated in Figure 4A and Table S4, a total of 1995 genes exhibited significant transcriptional alterations after NDV infection, comprising 1402 upregulated genes and 593 downregulated genes.

We conducted GO analysis on the 1995 differentially expressed genes. Functional annotation was performed from three perspectives: biological process, cellular component, and molecular function, and the results are illustrated in Figure 4B,C and Table S5. The upregulated genes were primarily involved in the defense response to the virus, the regulation of the viral process, and the cytokine-mediated signaling pathway. The downregulated genes were mainly implicated in the process of ribosome biogenesis as well as the RNA processing and metabolism. Additionally, we performed KEGG pathway analysis on the differentially expressed genes. As shown in Figure 4D,E and Table S6, the upregulated genes were predominantly enriched in the virus infection and a series of immune signaling pathways such as TNF signaling, RIG-I-like receptor signaling, NOD-like receptor signaling, and Toll-like receptor signaling, while the downregulated genes were mainly enriched in the pathways associated with DNA replication, cell cycle, and metabolic processes of fundamental biomolecules in life.

**Figure 5:**
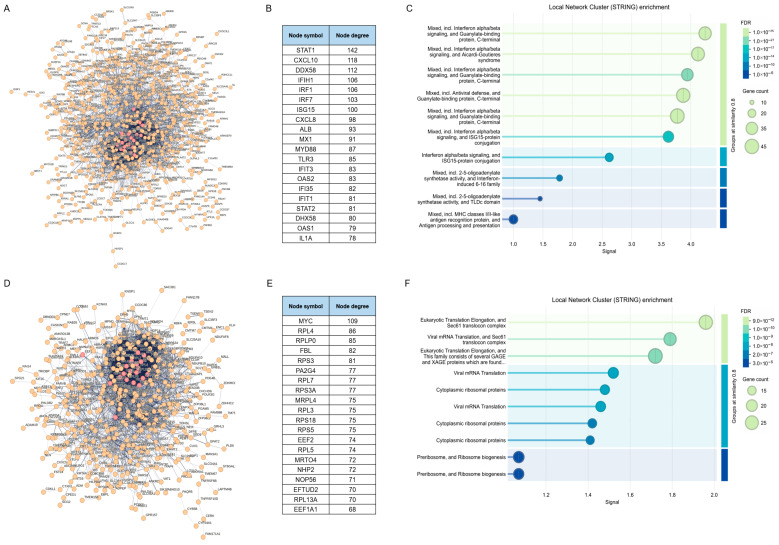
PPI analysis of the putative effectors involved in NDV oncolysis. (**A**,**B**) PPI networks of 776 upregulated genes with the top 20 ranked genes highlighted in red and listed separately. Proteins are represented as nodes with lines indicating functional relationship. (**C**) Local STRlNG network cluster enrichment analysis of 776 upregulated genes. (**D**,**E**) PPI networks of 436 downregulated genes with the top 20 ranked genes highlighted in red and listed separately. Proteins are represented as nodes with lines indicating functional relationship. (**F**) Local STRlNG network cluster enrichment analysis of 436 downregulated genes.

Finally, we conducted PPI network analysis on the differentially expressed genes to elucidate their interactions. As illustrated in Figure 5A, 776 out of 1042 upregulated genes exhibited associations with each other. The top 20 protein-coding hub genes primarily clustered around the IFN-signaling pathway and innate immune responses, functioning as follows: *DDX58* (RIG-I), *IFIH1* (MDA5), and *TLR3* are pattern recognition receptors which can recognize viral nucleic acids and initiate immune responses, meanwhile MYD88 serves as an adaptor protein in the process, activating the NF-κB and MAPK cascades to regulate the expression of pro-inflammatory genes; *CXCL8* and *CXCL10* are chemokines, playing pivotal roles in immune responses, inflammatory processes, cell migration, among others; *STAT1* and *STAT2* are key transcriptional factors in the IFN signaling pathway, which can induce the production of IFN-α/β and subsequently upregulate interferon-stimulated genes (ISGs)—including *ISG15*, *MX1*, *OAS1/2*, *IFIT1/3*, and *IFI35*–to suppress viral replication. Moreover, we conducted functional cluster analysis of the PPI networks, and, as shown in Figure 5C, these network clusters are significantly enriched in antiviral immune responses mediated by the IFN-signaling pathway, supporting the hypothesis that innate immune signaling was activated via pattern recognition receptors, leading to the secretion of IFNs and chemokines. Among the 593 downregulated genes, 436 genes were functionally associated with one another. The top 20 protein-coding hub genes primarily clustered around ribosome and protein biosynthesis as well as cell proliferation regulation: ribosomal proteins such as RPL4, RPLP0, RPS3, RPL7, RPS3A, RPL3, RPS18, RPS5, RPL5, and RPL13A are structural components of the large (RPL) and small (RPS) subunits of the ribosome, along with proteins FBL, NHP2, NOP56, and PA2G4, which are involved in rRNA modification and ribosome assembly, and they collectively facilitate the formation of the ribosome–RNA complex. MRT04 and EFTUD2 participate in RNA processing and ribosomal function, ensuring translational fidelity. *EEF2* and *EEF1A1* serve as translational elongation factors, facilitating peptide chain elongation and protein synthesis. *MYC* is a pivotal transcription factor that governs the expression of ribosomal proteins and synergizes with PA2G4 to promote protein synthesis and cell growth. Functional cluster analysis of the PPI networks revealed that these network clusters are significantly enriched in pathways associated with mRNA transcription, translation, and protein biosynthesis, in line with the results from PPI network analysis (Figure 5F).

### 3.4. Comprehensive Analysis of the Regulatory Mechanisms Involved in NDV Oncolysis

To further investigate the mechanism involved in the killing effect of NDV on HCT116 cells, we conducted integrated analysis of CRISPR/Cas9 knockout screening data and RNA-sequencing data at both protein function and transcriptional levels.

**Figure 6:**
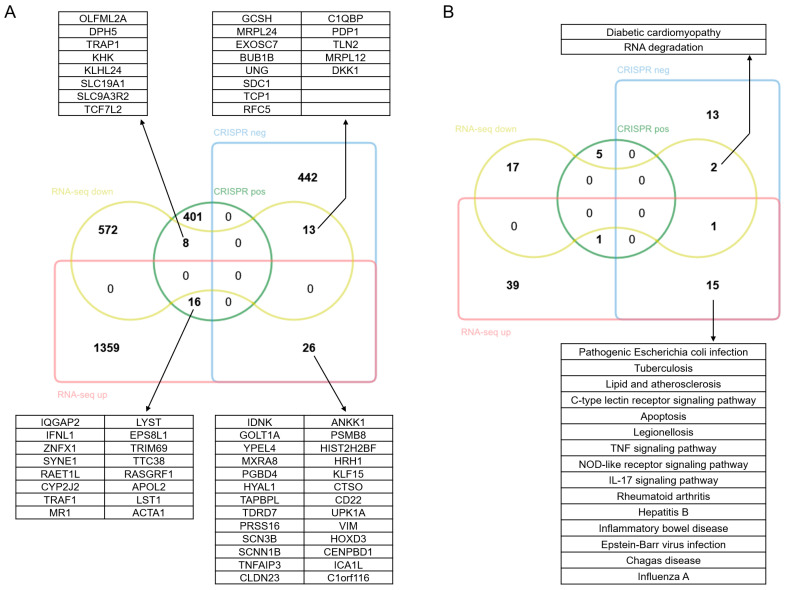
Venn diagrams. (**A**) Venn diagram depicting the differentially expressed genes identified via RNA-sequencing analysis and positive and negative selection genes from CRISPR/Cas9 knockout screening. (**B**) Venn diagram of KEGG pathways enriched using differentially expressed genes and positive and negative selection genes.

As illustrated in Figure 6A, among the 425 positive genes identified through CRISPR screening, 8 genes were significantly downregulated following NDV infection, while 16 genes were significantly upregulated. Among the downregulated genes, *KHK* is involved in glycolysis and lipid metabolism; *SLC19A1* mediates the uptake of folate, which is essential for viral nucleic acid synthesis; *DPH5* participates in ribosome synthesis; *OLFML2A* can modulate the activity of immune cells; *TCF7L2*, as a transcription factor in the Wnt pathway, regulates the expression of genes associated with cell proliferation. Among the upregulated genes, *ZNFX1* is capable of recognizing viral RNA and activating innate immune response; *TRAF1* serves as an adaptor protein in TNF receptor signaling, regulating the NF-κB and apoptosis pathways; *RAET1L* can activate NKG2D-mediated killing by NK cells; *IFNL1* may clear viruses by promoting apoptosis or enhancing viral recognition to activate downstream ISGs; *ACTA1* is involved in viral endocytosis or cytoskeletal remodeling. In our study, oncolytic effect of NDV was attenuated when positive selection genes were knocked out, suggesting these genes are key host regulatory factors responding to NDV infection. Additionally, since NDV infection inhibited the expression of genes related to metabolism and proliferation in HCT116 cells while promoting the expression of antiviral innate immunity-related genes, it is implied that NDV might exert oncolytic effect by inhibiting cell growth, and reducing the antiviral capacity of cells might further enhance NDV’s oncolytic efficacy.

As shown in Figure 6A, among the 481 negative genes identified through CRISPR screening, 13 genes were significantly downregulated following NDV infection, while 26 genes were markedly upregulated. Among the downregulated genes, *BUB1B* functions as a mitotic checkpoint protein; *RFC5* is involved in DNA repair; *GCSH*, a mitochondrial lipoic acid synthase, sustains mitochondrial function; *MRPL24* and *MRPL12* are mitochondrial ribosomal proteins; *SDC1*, a cell surface adhesion molecule, may hinder viral adsorption or invasion. Among the upregulated genes, *TNFAIP3* and *IDNK* play roles in suppressing inflammatory responses and apoptosis; *SCN3B* and *SCNN1B* are sodium channel subunits that regulate ionic homeostasis, and their deletion will lead to abnormal membrane potential, facilitating viral spread and lowering the threshold for cell lysis; *TAPBPL* mediates the interaction between MHC-I and the transporter associated with antigen processing; *PRSS16* participates in the positive selection of T cells, regulating the specificity of antigen presentation. Additionally, the upregulated gene list also includes several oncogenes, such as *YPEL4*, *HYAL1*, *CLDN23*, *PSMB8*, *UPK1A*, and *HOXD3*. In our study, the oncolytic effect of NDV was enhanced after knocking out the negative selection genes, suggesting these genes can effectively resist NDV infection or killing. In addition, NDV infection inhibits the expression of genes related to maintaining cell homeostasis in HCT116 cells and promotes anti-apoptosis, immune regulation, and tumor-related gene expression, indicating that NDV infection disrupts cell homeostasis, and the ensuing immune response and activation of anti-apoptosis and tumor-related genes may be the reason for the limited oncolytic effect of NDV on HCT116 cells.

We performed a similar analysis on the signaling pathways, and the results are shown in Figure 6B. Among the six signaling pathways enriched by positive selection genes, only NF-κB signaling was activated at transcriptional level upon NDV infection, while no significant changes were observed in the other pathways. This suggested that the activation of NF-κB signaling might be a prerequisite for NDV to exert its oncolytic effect on HCT116 cells. Among the 31 signaling pathways enriched by negative selection genes, 18 pathways were affected at transcriptional level upon NDV infection. Among these, 15 pathways were exclusively activated by NDV infection, all of which are related to viral infection and apoptosis signaling pathways, indicating that NDV infection triggers a broad antiviral immune response in HCT116 cells. Two of the 18 pathways, namely diabetic cardiomyopathy and RNA degradation, were exclusively repressed by NDV infection, indicating that NDV infection may suppress host cellular homeostasis-related pathways to facilitate viral replication and evade antiviral immunity, thereby creating a favorable microenvironment for viral replication and propagation.

## 4. Discussion

This study comprehensively explored the oncolytic mechanism of NDV against colon cancer cells through whole-genome CRISPR-Cas9 knockout screening and RNA sequencing technologies. The results revealed that NDV infection disrupts cell homeostasis, which exerts an oncolytic effect by inhibiting cell metabolism and proliferation. Meanwhile, the antiviral immune response triggered by NDV infection, along with the activation of anti-apoptotic signaling pathways, may be responsible for the limited oncolytic efficacy of NDV against HCT116 cells. These findings not only enhance our understanding of the oncolytic mechanism of NDV against colonic carcinoma but also provide novel strategies and targets for the development of NDV-based engineered oncolytic viruses.

The cell biological experiments confirmed that NDV has a significant oncolytic effect on colon cancer cells HCT116 but no obvious effect on normal colon epithelial cells NCM460. However, compared with other types of tumor cells, the oncolytic efficacy of NDV against HCT116 cells is relatively limited, which may be related to the specific strain of NDV that we used or determined by tumor heterogeneity [18]. To answer this question and enhance NDV oncolytic efficacy, we investigated the key host determinants and the transcriptional profiles of host effectors involved in NDV infection and killing, and through bioinformatic analysis, we further elucidated their potential regulatory relationships.

Through CRISPR-Cas9 knockout screening, we identified the positive selection genes, which were primarily implicated in biological processes such as amino acid and metal ion transport, as well as cellular responses to sterols, while the negative selection genes were mainly involved in the regulation of the NIK/NF-κB signaling pathway and cytoskeletal and membrane components, which are crucial for the cell’s defense mechanisms against viral infection [26]. In particular, the NIK/NF-κB pathway is a central regulator of inflammation and immune responses, and its activation leads to the production of a series of pro-inflammatory cytokines and antiviral factors [27]. Therefore, the presence of these negative selection genes suggests that they likely act to resist NDV infection, and their knockout may enhance cancer cell susceptibility to NDV—findings that align with the well-documented role of the NF-κB pathway in NDV-host interactions [28,29]. RNA sequencing results revealed changes in the gene expression profile of HCT116 cells following NDV infection. Upregulated genes were predominantly enriched in antiviral defenses and the cytokine-mediated signaling pathways, including TNF signaling, RIG-I-like receptor signaling, NOD-like receptor signaling, and Toll-like receptor signaling, pathways whose activation in response to viral RNA sensing during NDV–host interactions are well established [29]. Conversely, downregulated genes were primarily involved in biogenesis and metabolic processes. These findings, from a new perspective, provided evidence that NDV infection not only induces apoptosis and direct killing in tumor cells, but also enhances oncolytic efficacy by activating the host’s antiviral immune response, consistent with prior studies on oncolytic viruses [5,30]. Following PPI network cluster analysis further showed that the antiviral immune responses were mediated by the IFN signaling pathway, supporting the hypothesis that innate immune signaling (e.g., STAT1/2) was activated via pattern recognition receptors (e.g., DDX58, MDA5,and TLR3), leading to the secretion of IFNs and chemokines (e.g., CXCL8/10). Then this cascade induced antiviral ISG expression (e.g., ISG15, MX1, OAS1/2, IFIT1/3, and IFI35), recruited immune cells, and modulated inflammatory responses [31,32]. Modifications of NDV targeting these pathways hold potential to tailor the virus to counteract specific resistance mechanisms and thereby enhance its oncolytic efficacy. For instance, NDV has been engineered to express pro-apoptotic factors (e.g., TRAIL) or immunomodulatory cytokines (e.g., GM-CSF) to boost oncolytic activity and immune cell recruitment. A modified NDV engineered to encode TRAIL (NDV-TRAIL) exhibited increased apoptotic activity in colon cancer cells compared to wild-type NDV [33], while GM-CSF-expressing NDV drove robust oncolytic effects and durable systemic antitumor immunity in both human tumor cells and three syngeneic in vivo models [34]. Such biotechnological engineering strategies underscore the potential to customize NDV to counteract specific resistance pathways. Interestingly, although NDV infection induced apoptosis in HCT116 cells (Figure 1B–D), apoptosis-related signaling pathways were not significantly enriched in either our CRISPR screening or RNA-seq analyses. This discrepancy is consistent with prior reports: the HN protein of NDV induces apoptosis by triggering lysosomal membrane permeabilization [35], while the V protein inhibits apoptosis to promote viral replication via interactions with CacyBP/SIP [36,37,38]. Thus, other mechanisms, such as secondary cell death in neighboring uninfected cells induced by damage-associated molecular patterns (DAMPs) released from dying NDV-infected cells, cannot be ruled out.

Immunotherapy is currently the cutting-edge treatment for cancer, and to some extent, it is replacing the traditional chemotherapy and radiation therapy, which have significant side effects and poor specificity [39]. The mechanisms by which tumor immunotherapy works include the following: directly disrupting receptor–ligand signaling, impeding immune tolerance pathways in tumors and stimulating body’s immune system [40]. Currently, tumor immunotherapy approaches include monoclonal antibodies, small molecules, cytokines, oncolytic viruses, etc., and the advantage of oncolytic virus therapy is that it is highly specific to tumor cells and has little dependence on specific receptors [41,42]. In addition, oncolytic viruses can disrupt the tumor microenvironment-driven immune suppression and kill tumors by triggering and/or enhancing anti-tumor immune response [6,43]. T-VEC, an oncolytic virus product designed based on the herpes simplex virus, combines viral oncolysis with immunotherapy to produce synergistic therapeutic effects. The backbone of T-VEC is inserted with a gene fragment encoding GM-CSF to recruit and activate immune cells [7]. Clinical data have demonstrated that when T-VEC is administered to the tumor in melanoma patients, therapeutic effects can also be observed in the distal regions of non-injected lesions, indicating that T-VEC can induce the body to produce anti-tumor immune responses [44]. Nevertheless, to reach the goal of cure, further study and improvement are required.

Since the oncolytic ability of NDV was first confirmed in 1965, extensive research has demonstrated its efficacy against a broad spectrum of malignancies, including breast, ovarian, colorectal, pancreatic, renal, prostate, lung, and liver cancers [18,20]. However, its oncolytic potency and underlying mechanisms vary significantly across tumor types, underscoring the need for systematic investigations into NDV’s context-dependent oncolytic mechanisms [20]. Our present study suggests that NDV’s oncolytic effect in colon cancer cells involves multi-level regulation. On the one hand, NDV weakens antiviral defenses and growth of tumor cells by inhibiting the expression of genes related to metabolism and proliferation while promoting the expression of antiviral innate immunity-related genes. On the other hand, NDV enhances the oncolytic effect by activating key signaling pathways such as the NF-κB. In addition, the knockout of cell cycle checkpoint proteins and DNA repair factors may render cells more susceptible to NDV by inducing hyperproliferation or unresolved DNA damage, thereby enhancing the killing advantage of NDV against the continuously proliferating cancer cells. These findings lay a foundation for future mechanistic studies. Given the inherent molecular and phenotypic heterogeneity of human colorectal cancer, our findings, derived from the HCT116 cell line, may not fully capture the breadth of human colorectal cancer heterogeneity. To address this, further investigations using multiple preclinical models, including additional human colorectal cancer cell lines, such as HT-29 and SW480, and patient-derived tumor samples, are necessary to better recapitulate tumor heterogeneity and microenvironmental interactions, thereby strengthening the translational relevance of our work.

## Data Availability

RNA-seq raw data were uploaded to the National Center for Biotechnology Information database under the accession number GSE297244.

## Supplementary Materials

The following supporting information can be downloaded at: https://www.mdpi.com/article/10.3390/v17081043/s1. Figure S1: Cell cycle analysis at 24 h post NDV infection; Table S1: Significant genes identified by genome-wide CRISPR-based screen; Table S2: GO analysis of significant genes identified by genome-wide CRISPR-based screen; Table S3: KEGG pathway analysis of significant genes identified by genome-wide CRISPR-based screen; Table S4: Differentially expressed genes (DEGs) identified by RNA-sequencing; Table S5: GO analysis of DEGs identified by RNA-sequencing; Table S6: KEGG pathway analysis of DEGs identified by RNA-sequencing.
