# Supplementary figures and images for: Genome-Wide Screening Reveals the Oncolytic Mechanism of Newcastle Disease Virus in a Human Colonic Carcinoma Cell Line
Source: Viruses. 2025 Jul 25;17(8):1043. doi: 10.3390/v17081043 (PMC12390116; doi:10.3390/v17081043)

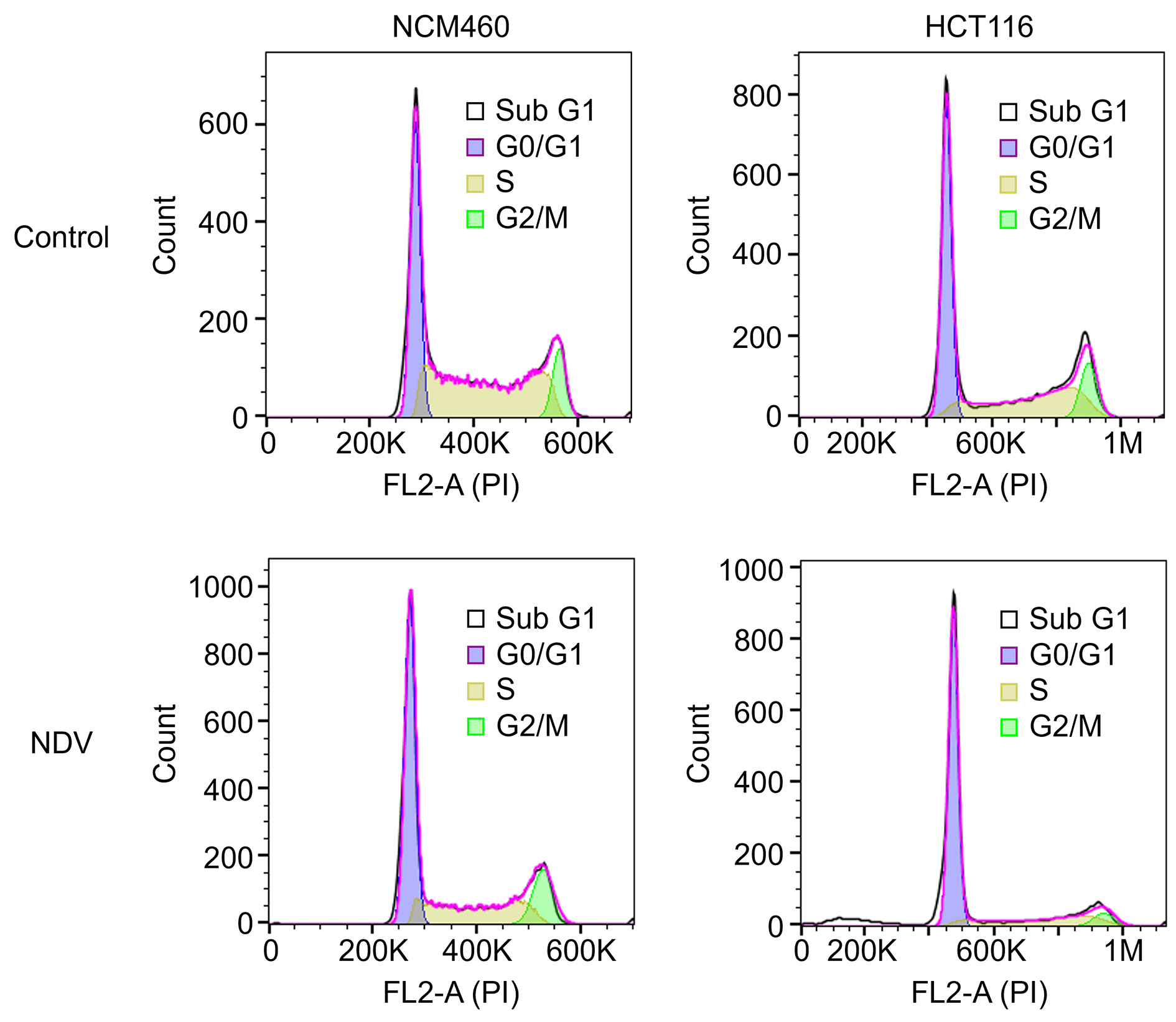

Supplement: Supplementary file 1 [file viruses-17-01043-s001.zip › Figure S1.tif]
